# Comparative genomics reveals new insights into the evolution of the IncA and IncC family of plasmids

**DOI:** 10.3389/fmicb.2022.1045314

**Published:** 2022-11-16

**Authors:** Fengwei Zhang, Xianwei Ye, Zhiqiu Yin, Mingda Hu, Boqian Wang, Wenting Liu, Beiping Li, Hongguang Ren, Yuan Jin, Junjie Yue

**Affiliations:** ^1^Medical College of Guizhou University, Guiyang, China; ^2^State Key Laboratory of Pathogen and Biosecurity, Beijing Institute of Biotechnology, Beijing, China; ^3^Guizhou Provincial People’s Hospital, Guiyang, China; ^4^National Engineering Research Center for Efficient Utilization of Soil and Fertilizer Resources, College of Resources and Environment, Shandong Agricultural University, Tai’an, China

**Keywords:** IncA/C group plasmid, comparative genomics, backbone, T4SS, mobile genetic element

## Abstract

Incompatibility groups IncA and IncC plasmids are of great concern due to their ability to disseminate antibiotic resistance in bacteria via conjugative transfer. A deep understanding of their genomic structures and evolutionary characteristics is of great significance for improving our knowledge about its multidrug-resistance evolution and dissemination. However, current knowledge of their backbone structure, features of core functional modules and the characteristics of variable regions is based on a few plasmids, which highlights the need for a comprehensive systematic study. The present study thoroughly compared and analysed 678 IncA and IncC plasmid genomes. We found that their core functional genes were occasionally deficient and sometimes existed as multiple functional copies/multiple families, which resulted in much diversity. The phylogeny of 13 core functional genes corresponded well to the plasmid subtypes. The conjugative transfer system gained diverse complexity and exhibited many previously unnoticed types with multiple combinations. The insertion of mobile genetic elements (MGEs) in plasmids varied between types and was present in 4 insertion spots in different types of plasmids with certain types of transposons, integrons and insertion sequences. The impact of gene duplication, deletion, the insertion of MGEs, genome rearrangement and recombination resulted in the complex dynamic variable backbone of IncA and IncC plasmids. And IncA and IncC plasmids were more complex than their closest relative SXT/R391 integrative conjugative elements (ICEs), which included nearly all of the diversity of SXT/R391 in key systems. Our work demonstrated a global and systematic view of the IncA and IncC plasmids and provides many new insights into their genome evolution.

## Importance

The incompatible plasmid groups IncA and IncC are of great concern for spread and dissemination of multi-drug resistant phenotypes among diverse bacterial species via conjugative transfer. A deep understanding of their genomic structures and evolutionary features based on large-scale plasmids sequences is of great significance for improving our knowledge about its multidrug-resistance evolution and dissemination. The present study showed their core functional genes and conjugative transfer system possess much diverse complexity than typical depicted. And the backbone of IncA and IncC plasmids were complex dynamic that were influenced by gene duplication and loss, the insertion of MGEs which varied between plasmids types and genomic insertion spots, genome rearrangement and recombination. Compared to their closest relative SXT/R391 ICEs, IncA and IncC plasmids included nearly all of its diversity in key systems. Our work demonstrated a global and systematic view of the IncA and IncC plasmids and revealed many previously unknown features and diversity.

## Introduction

Incompatible plasmids groups A (IncA) and C (IncC) have a broad host range, including *Salmonella enterica, Yersinia pestis,* and *Escherichia coli* etc., and are involved in the dissemination of antibiotic and mercuric ion resistance in Gram-negative bacteria (Harmer and Hall, 2015). Early studies demonstrated that entry exclusion made two plasmids from a group unable to coexist in a cell and recombination between IncA and IncC plasmids occurred frequently (Ambrose et al., 2018a). Therefore, IncA and IncC were combined into an IncA/C complex group for more than 40 years. Until recent years, an experimental research found that IncA and IncC plasmids were actually compatible, and IncA/C should be designated IncA and IncC separately (Harmer et al., 2017). Based on differences in the R1 and R2 regions, i1 and i2 fragments and a short patch in the genome, the IncC group were classified into type 1a, type 1b and type 2 (Harmer and Hall, 2014). While many plasmids gain the characteristics of multiple types and cannot be classified into any one type which suggests a limitation on current typing (Lei et al., 2017; Papagiannitsis et al., 2017; Ambrose et al., 2018b).

Early research found a shared IncA and IncC backbone between several plasmids which consisted of 135 syntenic genes and a complete backbone was also obtained by removing all mobile elements in 2014 (Welch et al., 2007; Harmer and Hall, 2014). An IncC plasmid, pSCEC2, was compared with five plasmids to determine the backbone structure, including replication and partial conjugation transfer system-related genes (Zhang et al., 2014). A study based on seven IncA or IncC plasmids found that the common backbone structure included genes related to replication, conjugation transfer and plasmid maintenance systems (Ye et al., 2016). Another study based on the genomic sequences of eight IncA or IncC plasmids found that the genes shared by these plasmids included genes attributed to replication, conjugative transfer, recombination modules and some DNA metabolism and other module-related genes (Li P. et al., 2018). These distinctions highlight the necessity of executing detailed examinations of the plasmid backbone based on large-scale IncA and IncC plasmids.

Conjugative transfer, which mediates DNA and macromolecule protein transfer between two bacterial cells *via* direct contact, is the most efficient mechanism of horizontal gene transfer (De La Cruz et al., 2010; Poulin-Laprade et al., 2015). For transmissible plasmids, such as IncA and IncC, conjugation is executed *via* a conjugative transfer system that includes mobility (MOB) and mating pair formation (MPF) genes (Smillie et al., 2010; Álvarez-Rodríguez et al., 2020). MOB consists of the original region of transfer, a relaxase gene, type IV coupling protein (T4CP) gene and any auxiliary proteins (Burrus, 2017). MPF in IncA and IncC plasmids is a type IV secretion system (T4SS), which is an envelope-spanning structure that transfer nucleotides or effected proteins to the outside of the cell (Costa et al., 2021). The T4SSs in IncA and IncC plasmids are generally depicted as three gene clusters: *traLEKBVA*, *traCWUN*, and *traFHG* (Guglielmini et al., 2013). However, studies found that plasmid pCFSAN007405 did not carry *traCWUN*, and pCFSAN007405 had extra conjugative transfer genes, such as *traA*, *traV* and *traB,* which were located in different orders compared to the *traBVA* gene cluster (Hoffmann et al., 2017). All of these findings suggest the complexity of the conjugative transfer system, and the reason underlying these phenomena requires further exploration.

Many mobile genetic elements (MGEs), such as integrons (In), transposons (Tn) and insertion sequences (IS), exist in IncA and IncC plasmids. Plasmid pYR1 carried a putative 6.8 kb transposon inserted into an ORF with an unknown function (Welch et al., 2007). Class I integrons frequently occurred in plasmid pUO-STmRV1 (Vázquez et al., 2021). MGEs also provide adaptive traits to hosts in particular conditions, such as antibiotic resistance and resistance to heavy metals and phage infection (Welch et al., 2007; Fricke et al., 2009; Wozniak and Waldor, 2009; Harmer et al., 2017; Lei et al., 2018). For example, antibiotic resistance islands (ARIs), such as ARI-A, ARI-B and ISEcp1-*bla_CMY-2_*, are MGEs embedded in other transmissible or integrated elements that encode clusters of antibiotic resistance genes, which are frequently disseminated *via* horizontal gene transfer (Ambrose et al., 2018b). Despite these findings, current knowledge of different types of mobile genetic elements in IncA and IncC plasmids is limited to few plasmid comparisons, and systematic studies are lacking.

The closest known relative to the IncA and IncC plasmids, the integrative conjugative element (ICE) SXT/R391, harbour highly similar and almost syntenic core sets of conserved genes with IncA and IncC plasmids (Wozniak and Waldor, 2009). The line between conjugative plasmids and ICEs may be getting thinner (Carraro et al., 2015; Hall et al., 2022). However, the regulatory systems between these factors are divergent in phylogenetic relationships (Poulin-Laprade et al., 2015), including obviously different replication mechanisms. A thorough comparison would provide more knowledge of their similarities and differences in diversity.

With the introduction of PCR-based replicon typing (PBRT; Russo et al., 2022), more IncA and IncC plasmids were identified in bacterial species. This study used available IncA and IncC plasmid sequences to revise core functional gene sets and backbone evolution and comprehensively characterised the essential modules of these plasmids, such as the conjugative transfer system and all MGEs embedded in comparison with SXT/R391. Our study provides a comprehensive view of IncA and IncC plasmid genome evolution and valuable new insight into their complex characteristics.

## Results

### IncA and IncC plasmids included in this analysis

**Figure 1:**
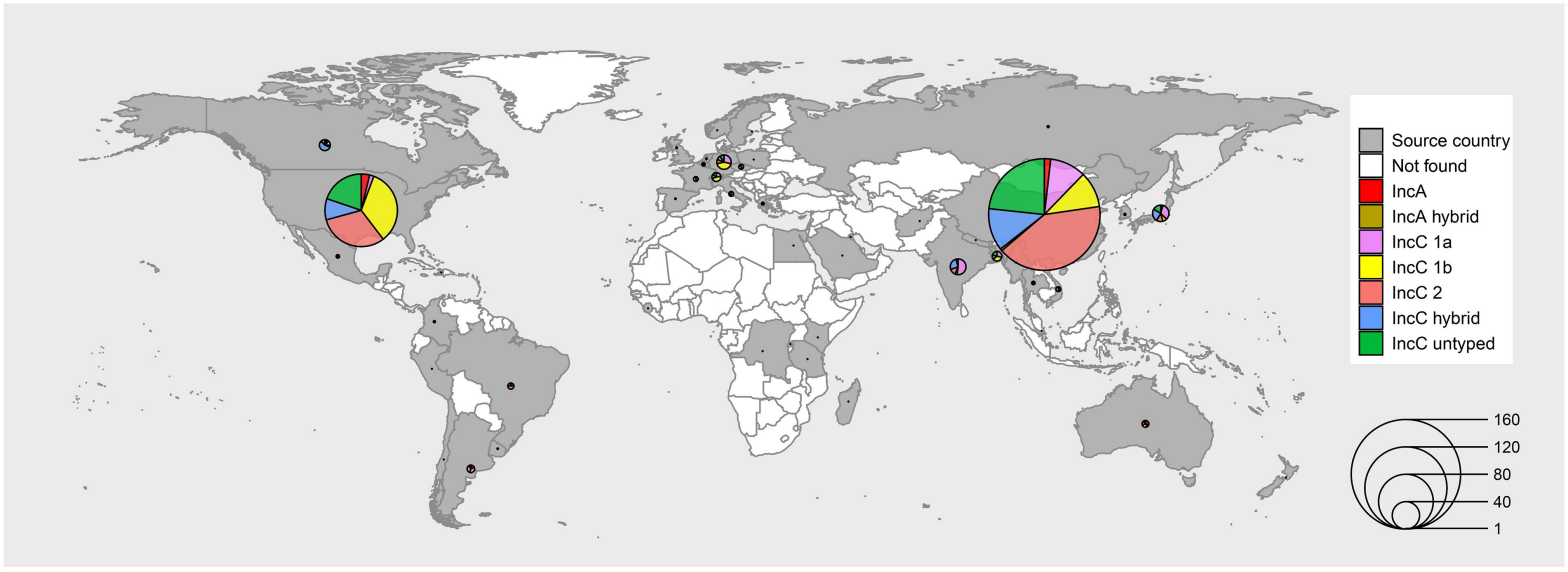
Sources and types of IncA and IncC plasmids in our dataset. The regions with grey colours represent that plasmids have been isolated, and white represents that no plasmid was isolated. The size of the circles in the map indicates the plasmid number. The pie charts with different colours show the proportion of types of plasmids isolated from different countries.

We manually curated the genome sequences and meta-information of 678 plasmids from the sequence databases, including 33 IncA plasmids, 3 hybrid-type IncA plasmids, 93 type 1a IncC plasmids, 110 type 1b IncC plasmids, 206 type 2 IncC plasmids, 145 untyped IncC plasmids and 88 hybrid-type IncC plasmids (Figure 1; Supplementary Table 1). The plasmids included in our analyses were isolated from 46 countries on 6 continents. Most plasmids originated from China and America, and the primary types were type 2 IncC and type 1b IncC, respectively (Figure 1). The year of collection was as early as 1905 and spanned more than 11 decades. Although the first IncC plasmid was isolated in the 1960s, most of the IncA and IncC plasmids which had collection data were collected after 2010 and accounted for about 54.1% of our data set. The plasmids were derived from 53 different hosts, including *Klebsiella pneumoniae*, *Escherichia coli* and *Salmonella enterica* (Supplementary Table 1).

### The core functional genes of IncA and IncC plasmids are occasionally deficient and sometimes exist as multiple functional copies/multiple families

**Figure 2:**
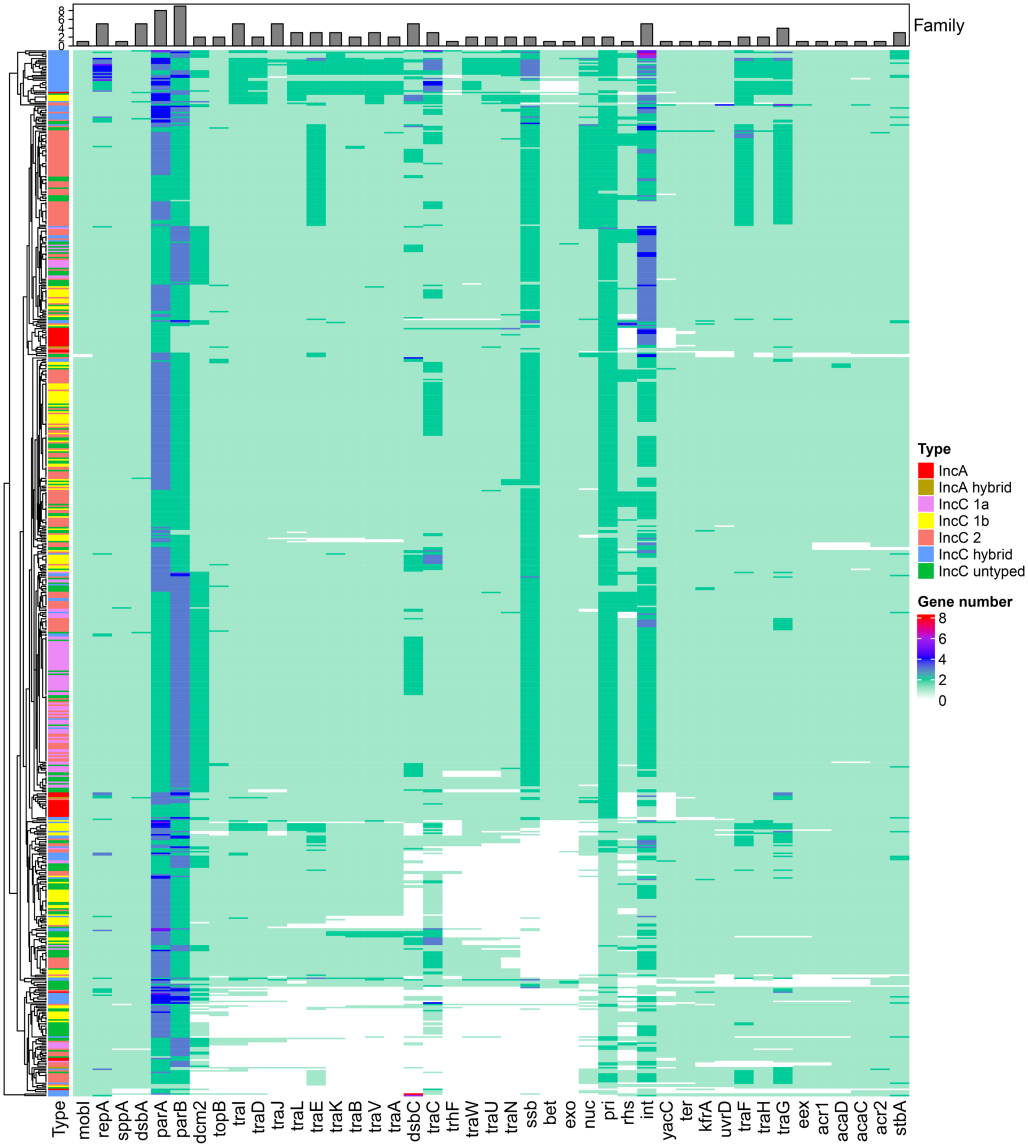
The distribution of core functional genes in plasmids. The heatmap represents the presence, absence, and abundance of core functional genes in different types of IncA and IncC plasmids. The bar plot on the top shows the family amount for genes.

The backbone genes of IncA and IncC plasmids were commonly considered to consist of 45 essential core genes involved in plasmid maintenance, regulation, replication, DNA metabolism, conjugative transfer and other functions (Harmer and Hall, 2015; Poulin-Laprade et al., 2015; Humbert et al., 2019). Since *dcm1* and *dcm3* were lacking in 431 and 226 plasmids respectively, we removed them from the core functional genes. Figure 2 shows the distribution of these core functional genes in all 678 plasmids. Notably, only *repA* which involved in replication was strictly conserved (i.e., present in all plasmids) and just plasmid pHS08-175-1 had the deletion of genes involved in plasmid maintenance (*parA* and *parB*). 414 of these 678 plasmids comprised all 43 core functional genes. There were 79 plasmids had a large portion of missing core functional genes, as shown by the empty regions of the heatmap in Figure 2. Many of these deletion events appeared in the conjugative transfer system (genes *traIDJLEKBVACWUNFHG*) and recombination (genes *ssb-bet-exo*) module, which should be necessary for DNA or macromolecule transfer and plasmid recombination. We also found multiple copies of core functional genes related to partition (*parA, parB*), DNA metabolism (*int, pri, ssb*) etc. in many plasmids (Figure 2). The protein clustering showed most of these multiple functional copy genes belonged to different families, which included replication module genes (*repA*), plasmid maintenance module genes (*parA*, *parB*, and *stbA*) and DNA metabolism module genes (*int, topB, ssb* and *nuc*) etc., which suggested that they harboured a variety of diversity (Supplementary Table 2).

### The phylogenetic relationship of a few core functional genes corresponded well to IncA and IncC plasmids typing

**Figure 3:**
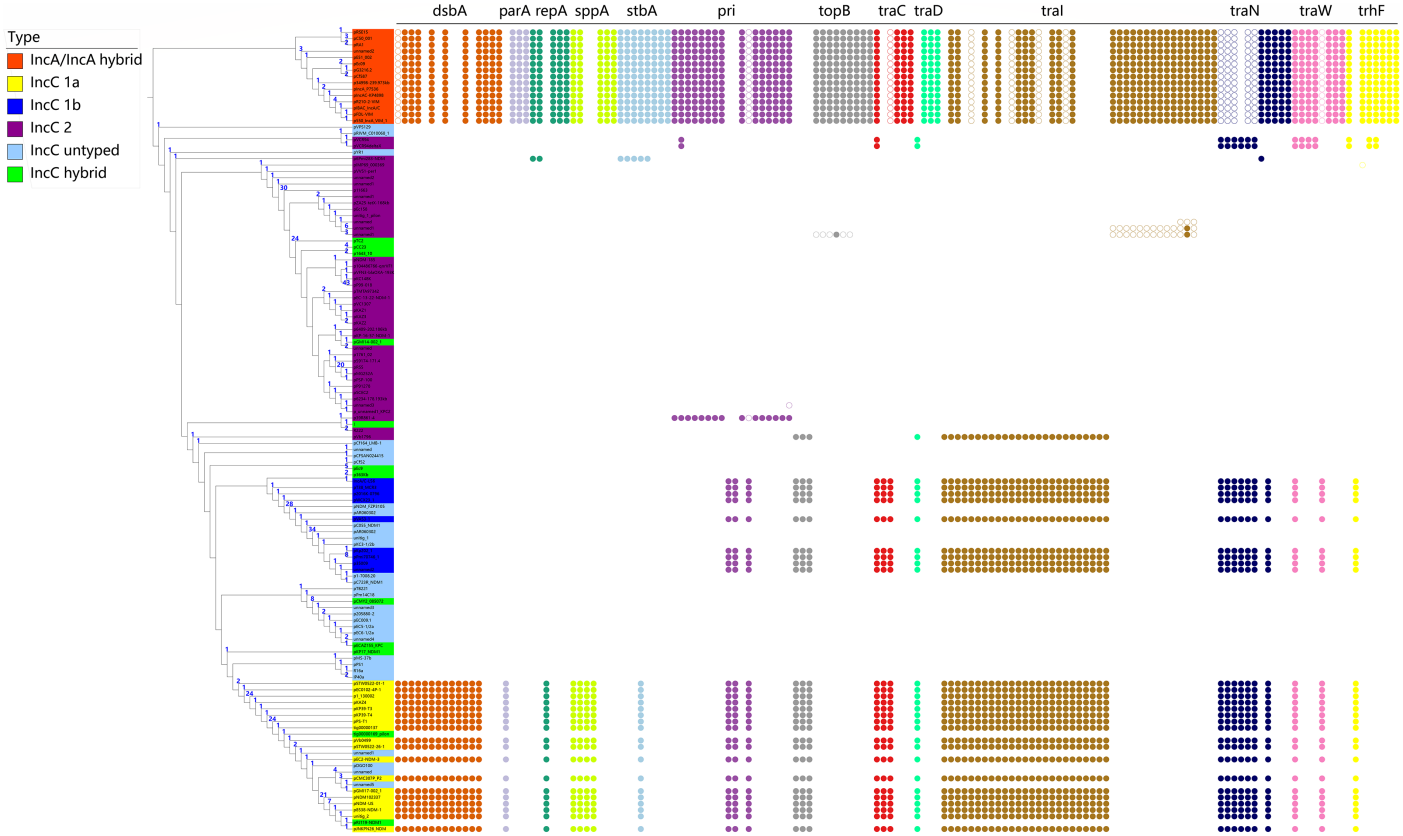
Phylogenetic relationship of representative plasmids of IncA and IncC plasmids and corresponding amino acid divergence. The evolutionary history was inferred using concatenated core functional genes. The difference in amino acids compared with the reference plasmid pR55 is shown by coloured circles that represent different amino acids.

We used core functional genes to construct the genealogy of IncA and IncC plasmids and found that the phylogenetic trees of 13 genes coincided well with the type of IncA and IncC plasmids (Figure 3; complete scenario in Supplementary Figure 1). The concatenating phylogenetic tree of 13 core functional genes clearly separated IncA, type 1a IncC, type 1b IncC and type 2 IncC plasmids into different clusters. The untyped IncC plasmids pMS-37b, pPS1 and IP40a etc. comprising orf1832 and *rhs1,* which are features of type 1 IncC, were clustered within the type 1 IncC group. The hybrid IncC plasmids p1643_10 and pTC2 were most closely related to the type 2 IncC branches, which is consistent with their genomic structure that contained characteristic regions of type 2 IncC plasmids. These phenomena suggested that these core functional gene phylogeny revealed the intrinsic type of these untyped or hybrid plasmids. We used pR55 as a reference to analyse the sequence divergence of these core functional genes and found that a single gene partially distinguished the types of plasmids (Figure 3; Supplementary Figures 2–4). As Figure 3 shows, the amino acids of the *topB*, and *pri* genes involved in DNA metabolism, the *traI*, *traD*, *trhF*, *traC*, *traW*, and *traN* genes of the conjugative transfer system were different at some loci between the IncA, type 1 IncC and type 2 IncC plasmids or IncA and IncC plasmids but almost identical between the type 1a and 1b plasmids. Therefore, these genes classified IncA, type 1 IncC and type 2 IncC plasmids but did not further distinguish type 1a from type 1b. The amino acids of *repA* related to replication, *parA, stbA* of the DNA maintenance module and *sppA* and *dsbA* were divergent in some loci between IncA, type 1a IncC and the union of type 1b and 2 IncC or IncA and type 1a IncC or IncA and the union of type 1b and 2 IncC plasmids. However, the sequences encoded by these genes in type 1b and type 2 IncC plasmids were nearly identical and made differentiation difficult (Figure 3). These results showed that some core genes were conserved in particular types of plasmids but showed amino acid differences in other types of plasmids. Our results indicated that the use of phylogeny and sequence divergence for these core functional genes provided an important supplementary perspective for plasmid classification and optimized the traditional typing method based on the presence or absence of featured regions.

### Conjugative transfer systems in IncA and IncC plasmids possess great diversity beyond the major type

The conjugative transfer system is an essential part of the backbone participating in the horizontal transfer of nucleotides and macromolecules. We systematically predicted and classified the conjugative transfer system by thoroughly searching conjugative-related genes in all plasmids (Table 1; Supplementary Table 3). As our results showed, except for the MOB_H_ subtype of the MOB module according to relaxase classification, which previous studies reported to be broadly distributed in IncA and IncC plasmids, subtypes such as MOB_C_, MOB_F_, MOB_P_, and MOB_Q_, also existed in these IncA and IncC plasmids. Among 619 plasmids containing relaxase genes, 607 harboured MOB_H_ encoded by the *traI* gene, and 12 plasmids contained some of MOB_C,_ MOB_F,_ MOB_P,_ MOB_Q_ and Other types of relaxases instead of MOB_H_. There were 158 plasmids comprised of more than one relaxase, other than MOB_H,_ 88 containing an additional MOB_C_ relaxase, 20 containing an additional MOB_F_ (Supplementary Table 3). Table 1 shows the summarised types of T4CPs in all plasmids. 619 of the 623 plasmids containing T4CPs contained the TrwB/TraD group. 120 of these plasmids contained two T4CPs, of which 9 plasmids had VirD4/TraG and TrwB/TraD group T4CPs and 111 plasmids harboured two TrwB/TraD group T4CPs. In addition, 3 plasmids contained three TrwB/TraD group T4CPs. Notably, only 4 plasmids containing MOB_P_-type and other relaxases except MOB_H_-type, these four plasmids also harboured single T4CP of VirD4/TraG group, as illustrated in Table 1. The MOB_H_ relaxase encoded by *traI* and TrwB/TraD group T4CP encoded by *traD* generally co-occurred in plasmids. The MOB_P_ relaxase always co-occurred with VirD4/TraG T4CP (Table 1; Supplementary Table 3). These results suggest a correlation between relaxases and T4CPs during the evolutionary process of IncA and IncC plasmids. Similar correlations were supported by findings that the phylogenies of relaxases and T4CPs were highly congruent in MOB_F,_ MOB_H,_ MOB_C,_ MOB_Q,_ MOB_P1_ relaxase-containing plasmids (Garcillán-Barcia et al., 2009).

**Table 1:** The types of conjugative transfer system components of plasmids containing T4CP.

Plasmid number^a^	Conjugative type^b^	Relaxase	T4CP	T4SS gene type
301	Conj	MOBH	TrwB/TraD	F_Tra
82	Conj	MOBH	TrwB/TraD	F_Tra,P_Trb
56	Conj	MOBH,MOBC	TrwB/TraD	F_Tra
43	Conj	MOBH	TrwB/TraD,TrwB/TraD	F_Tra,P_Trw
25	Conj	MOBH,MOBC	TrwB/TraD,TrwB/TraD	F_Tra,P_Trw
16	Conj	MOBH,Other	TrwB/TraD	F_Tra
15	Conj	MOBH,MOBF	TrwB/TraD,TrwB/TraD	F_Tra
14	mob_unconj	MOBH	TrwB/TraD	F_Tra
8	unmob	-	TrwB/TraD	F_Tra,P_Trw
7	Conj	MOBH	TrwB/TraD,TrwB/TraD	F_Tra,P_Trb,P_Trw
4	Conj	MOBH,MOBC	TrwB/TraD,TrwB/TraD	F_Tra,P_Trb,P_Trw
4	Conj	MOBH,MOBP	TrwB/TraD,VirD4/TraG	F_Tra,P_VirB
4	Conj	MOBF	TrwB/TraD	F_Tra
4	Conj	MOBH,Other	TrwB/TraD,TrwB/TraD	F_Tra
2	Conj	MOBH,MOBF	TrwB/TraD,TrwB/TraD	F_Tra,P_Trb
2	Conj	MOBH,MOBC,MOBC	TrwB/TraD	F_Tra
2	Conj	MOBH,MOBF,Other	TrwB/TraD,TrwB/TraD	F_Tra,P_Trb
2	Conj	MOBH,MOBC,Other	TrwB/TraD,TrwB/TraD	F_Tra
2	Conj	MOBH,MOBF	TrwB/TraD	F_Tra
2	Conj	MOBH,MOBC	TrwB/TraD	F_Tra,P_Trb
2	Conj	MOBH,MOBP,MOBP,MOBQ	TrwB/TraD,VirD4/TraG	F_Tra,I_Tra
1	Conj	MOBH,MOBF	TrwB/TraD,TrwB/TraD,TrwB/TraD	F_Tra,P_Trw
1	Conj	Other	TrwB/TraD,TrwB/TraD	F_Tra,P_Trw
1	Conj	MOBH,Other,Other	TrwB/TraD,TrwB/TraD	F_Tra
1	mob_unconj	MOBH,Other	TrwB/TraD	F_Tra
1	Conj	MOBH,MOBF,MOBP	TrwB/TraD,VirD4/TraG	F_Tra,P_Trb,P_VirB
1	Conj	MOBH,MOBF,MOBP	TrwB/TraD,VirD4/TraG	F_Tra,P_VirB
1	mob_unconj	MOBH	TrwB/TraD	F_Tra,P_Trw
1	Conj	MOBH,MOBH	TrwB/TraD,TrwB/TraD	F_Tra
1	Conj	MOBH,MOBC,Other,Other	TrwB/TraD,TrwB/TraD	F_Tra
1	Conj	MOBP,MOBP,MOBQ	VirD4/TraG	F_Tra,I_Tra
1	Conj	MOBF,MOBP	VirD4/TraG	F_Tra,P_VirB
1	Conj	MOBH,MOBC	TrwB/TraD	F_Tra,P_VirB
1	mob_unconj	MOBP,MOBP	VirD4/TraG	I_Tra
1	Conj	MOBH,MOBC,Other	TrwB/TraD,TrwB/TraD,TrwB/TraD	F_Tra,P_Trw
1	Conj	MOBH	TrwB/TraD,TrwB/TraD	F_Tra
1	Conj	MOBH	TrwB/TraD	F_Tra,P_VirB
1	Conj	MOBH,MOBF,Other	TrwB/TraD,TrwB/TraD	F_Tra
1	mob_unconj	MOBP,MOBP	VirD4/TraG	F_Tra,I_Tra
1	Conj	MOBH,MOBC,MOBP	TrwB/TraD,VirD4/TraG	F_Tra,P_VirB
1	Conj	MOBH,MOBC,MOBF	TrwB/TraD	F_Tra
1	mob_unconj	MOBH,MOBH	TrwB/TraD	F_Tra
1	Conj	MOBH,MOBQ	TrwB/TraD	F_Tra
1	Conj	MOBH	TrwB/TraD	F_Tra,P_Trw
1	Conj	MOBF	TrwB/TraD	F_Tra,P_Trw,P_VirB
1	Conj	MOBH,MOBC,MOBF	TrwB/TraD,TrwB/TraD,TrwB/TraD	F_Tra,P_Trw
1	Conj	MOBH,Other	TrwB/TraD,TrwB/TraD	F_Tra,P_Trb

a. The number of plasmids that have identical types of relaxases, T4CPs and T4SS genes.

b. Conj, conjugative plasmid; mob_unconj, mobilizable plasmid; unmob, non-mobilizable plasmid.

**Figure 4:**
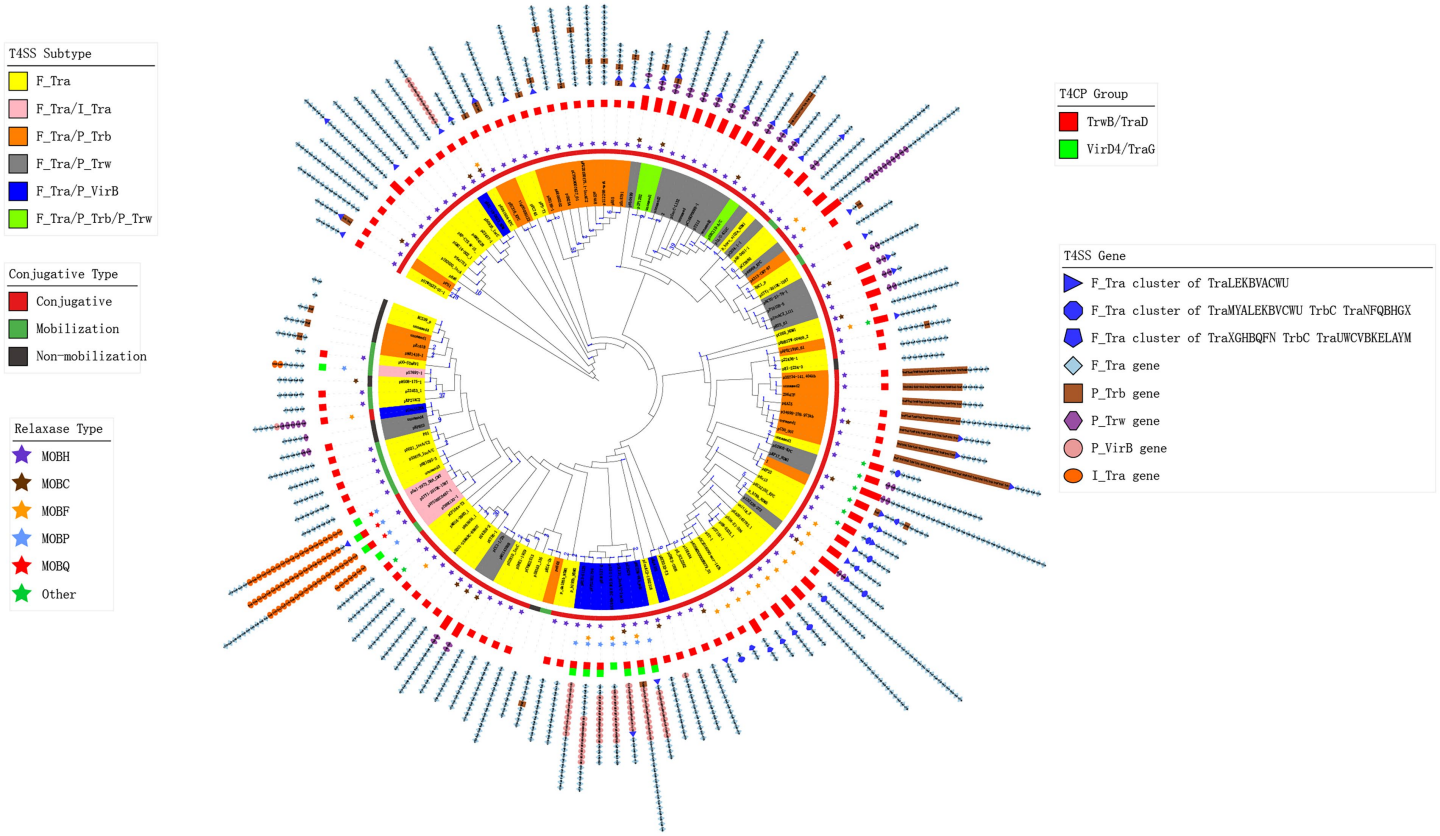
Gene content tree of the plasmid conjugative transfer system and its component types. The tree was constructed using the relaxase, T4CP and T4SS genes from representative plasmids. The number of plasmids represented by each node is marked on the branch. The colourful leaves represent the types of T4SS genes in plasmids. The conjugative types of the representative plasmids are shown by the strip plot. The types of relaxase genes are indicated by a star plot. The bar plot shows the types of T4CP genes in the plasmids. The concatenated colourful shapes represent the different types of T4SS genes in the plasmid.

For the MPF/T4SS module, we found that despite the three known T4SS gene clusters that belong to F-type T4SS, type P and type I T4SS-related genes existed. There were 204 plasmids containing P-type MPF/T4SS genes, and 4 plasmids contained I-type genes in addition to known F-type gene clusters and only pTHNK130-1 just had I-type T4SS. These different types of genes constituted a total of 129 combination classes of MPF/T4SSs gene clusters in all analysed plasmids (Figure 4). Figure 4 and Supplementary Table 3 show the constitution of representative MPF/T4SSs gene clusters of each class and its gene content tree. The MPF/T4SSs gene content tree shows that 278 plasmids contained the same F-type gene cluster of *traLEKBVACWUNFHG-Orf169,* and 190 plasmids lost some of the F-type genes (Figure 4). Ninety-three of the P-type gene-containing plasmids harboured the gene cluster *trwFE*, and nine had complete or partial clusters of *traFG-trbBCDEJLFGI*. Notably, plasmids pKP13e, and p35734-141.404 kb contained MOB_H_-type relaxase, TrwB/TraD-type T4CP and the partial F-type gene cluster *traFHG-Orf169* (Supplementary Table 3). However, p35734-141.404 kb was a conjugative plasmid, but pKP13e was a mobilizable plasmid according to a predictive method (Che et al., 2021; Figure 4; Table 1). The difference was that plasmid p35734-141.404 kb had an additional P-type gene cluster of *traFG-trbBCDEJLFGI*. This phenomenon suggests a functional compensation between F-type and P-type MPF/T4SS genes. In addition, in some plasmids, other relaxases replaced MOB_H_-type, which may also indicate a functional compensation between different types of relaxases. However, whether this relationship is present must be re-examined using modern molecular methods. All of these findings suggested that although the MPF/T4SS genes in most plasmids primarily belonged to the F-type, the composition of the conjugative transfer system in IncA and IncC plasmids was far more diverse and variable.

### The backbone of IncA and IncC plasmids is complex and dynamic rather than static

**Figure 5:**
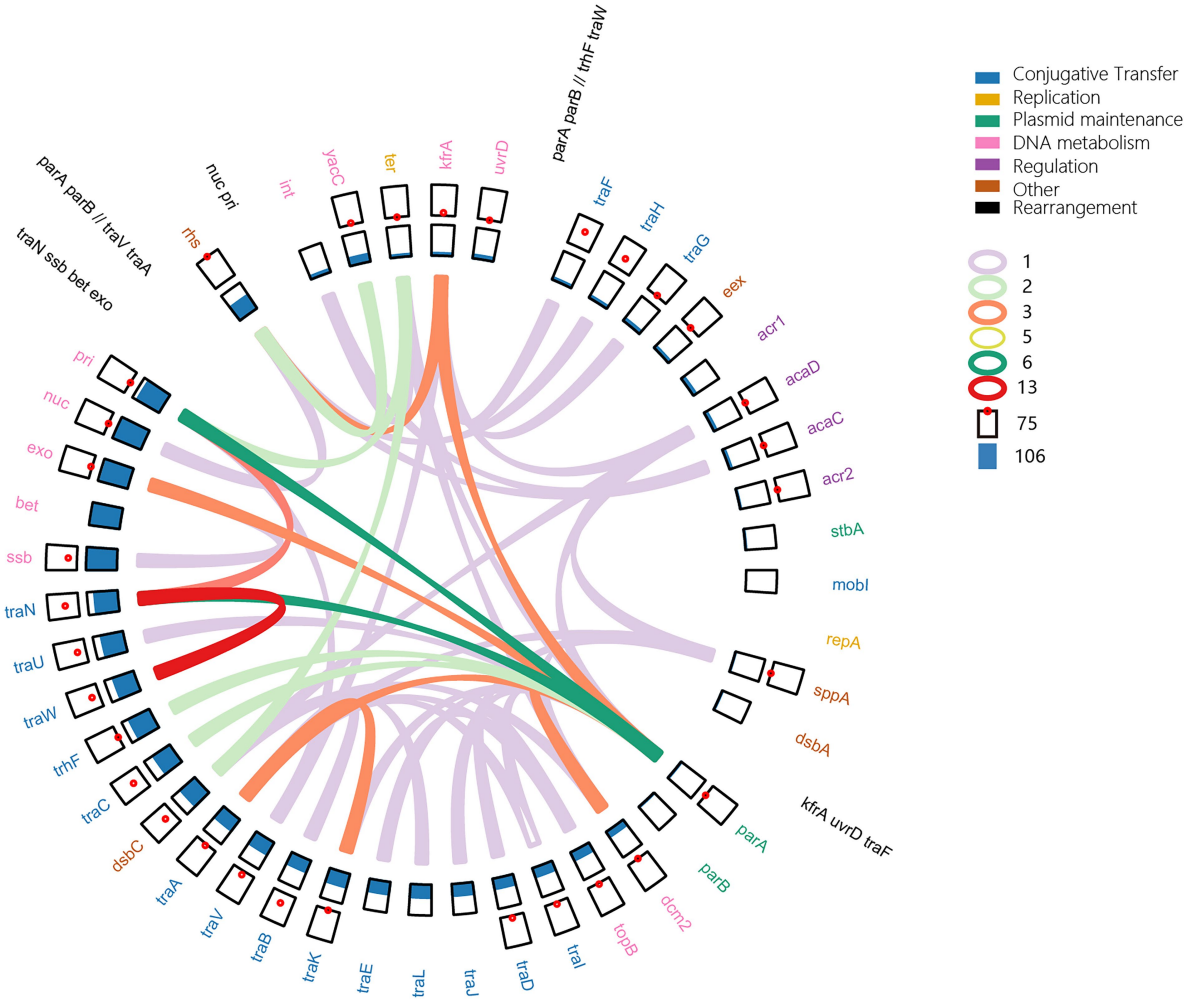
Dynamic structure of the IncA and IncC plasmid backbones. In the outside ring, the black gene clusters show the rearrangements. In the second ring, the coloured genes represent the shared backbone and the function of genes. All of the point plots and bar plots represent the number of genes with duplication and deletion in plasmids, respectively. The coloured arcs indicate that the gene fragments are inverted clockwise from the start genes to the end genes.

Although a common backbone was described in IncA and IncC plasmids (Poulin-Laprade et al., 2015), we did not find a conserved backbone in all 678 plasmids. Many core functional genes were lost in some plasmids, as shown in Figure 2. A common genomic structure was only found in 265 IncC plasmids (Supplementary Table 4). As Figure 5 shows, the shared backbone consists of essential functional genes involved in replication, conjugative transfer, plasmid maintenance, DNA metabolism, regulation and other functions. However, the backbone structures in the other plasmids were versatile due to different evolutionary events, such as gene loss and duplication, genomic inversion and rearrangement (Figure 5). For example, the gene cluster *traIDJLEKBVA* and genes *bet* and *exo* were deleted in 57 and 181 plasmids, respectively, and resulted in gene deficiency backbones (Figures 2, 5; Supplementary Table 4). Single gene or gene cluster duplication also occurred in the plasmids, such as a duplicate *traC* located in the region between *traA* and *dsbC* and a cluster of *traBWUN* in the interval of *rhs* and *int*, which was distributed in the region in front of *rhs* (Supplementary Table 4). The backbone in 77 plasmids exhibited genomic inversions, which included regions *traW-traN, parA-pri, parA-exo, parA-traN, parA-dcm2, rhs-kfrA* etc. For example, the genomic region from *parA* to *traA* in pN13-01070-1, pN13-01125 and p22429 plasmids were in the reverse order and the fragment from *parA* to *pri* in p21978-1, pCFSAN007427_01, p39R861–4, pEC6-1/2a, pCMY and p35189-1 was reversed compared to pR55 (Figure 5; Supplementary Table 4). The *parA-traA* region in plasmid p11298-tetA translocated to the region between *pri* and *rhs*. In pT5282-Ct2, the fragment of *kfrA-traF* translocated to the region between *dsbA* and *parA* in the reverse order, which resulted in backbone rearrangement (Figure 5; Supplementary Table 4). These findings suggested a complex variable and dynamic backbone of IncA and IncC plasmids.

### Insertion of MGEs varies by type and impacts plasmid gene evolution

**Figure 6:**
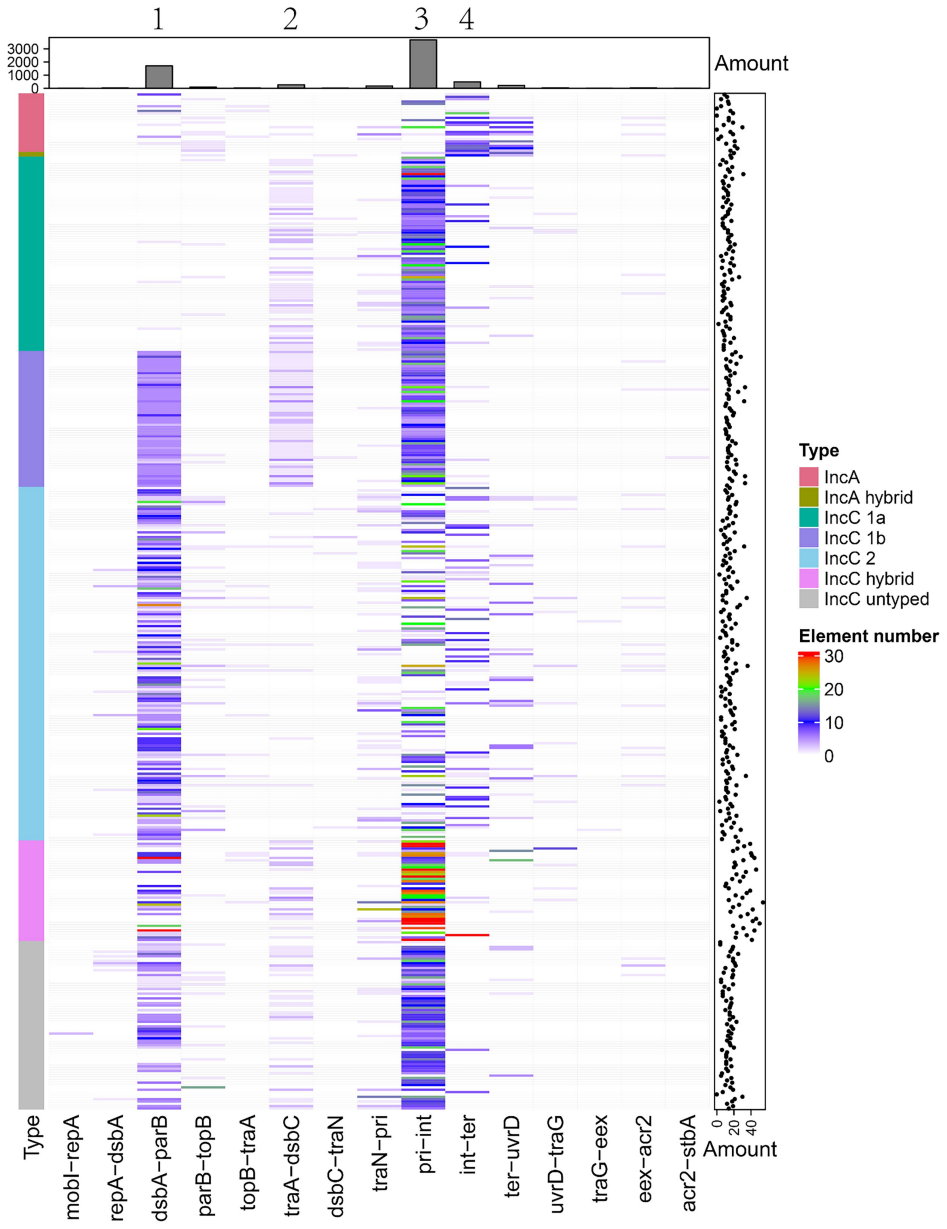
The distribution of insertion spots of IncA and IncC plasmids. Heatmap shows the presence and absence situations of mobile genetic elements (MGEs) in different types of plasmids. Both the bar charts and the scatter plots show the number of MGEs.

The IncA and IncC plasmids also contain variable DNA regions with many MGEs and antibiotic resistance genes, which may influence the genomic structure and phenotype. We predicted insertion sequences, integrons and transposons in all analysed plasmids. Figure 6 shows the distribution of these MGEs in plasmids. If a genome region comprised of MGEs was present in over half of a certain type of plasmid, we regarded it as a insertion spot (Figure 6). As Figure 6 shows, 4 insertion spots were found, but additional MGEs also existed in regions outside these insertion spots. Our results showed that the distribution of insertion spots exhibited a favoured relationship with plasmid types. For example, insertion spot 1 was dominant in type 1b and type 2 IncC, with 100.00, 92.05% of plasmids comprised of MGEs, respectively. Insertion spot 1, which contained the region of ARI-B, was generally deficient in type 1a IncC plasmids, which is consistent with the deletion of ARI-B in type 1a IncC plasmids. Insertion spot 2 primarily existed in type 1a and 1b IncC plasmids, in which 63.86 and 98.28% of plasmids had MGEs insertions, respectively (Supplementary Table 5). Insertion spot 3 mainly existed in type 1a, type 1b and 2 IncC plasmids with 98.8, 100.00 and 50.33% of plasmids having MGEs insertions, respectively. Insertion spot 4 was only present in IncA plasmids, which had insertions of MGEs in 64.00% of plasmids. Insertion spot 1 and 3 existed in most hybrid and untyped IncC plasmids (Supplementary Table 5).

**Figure 7:**
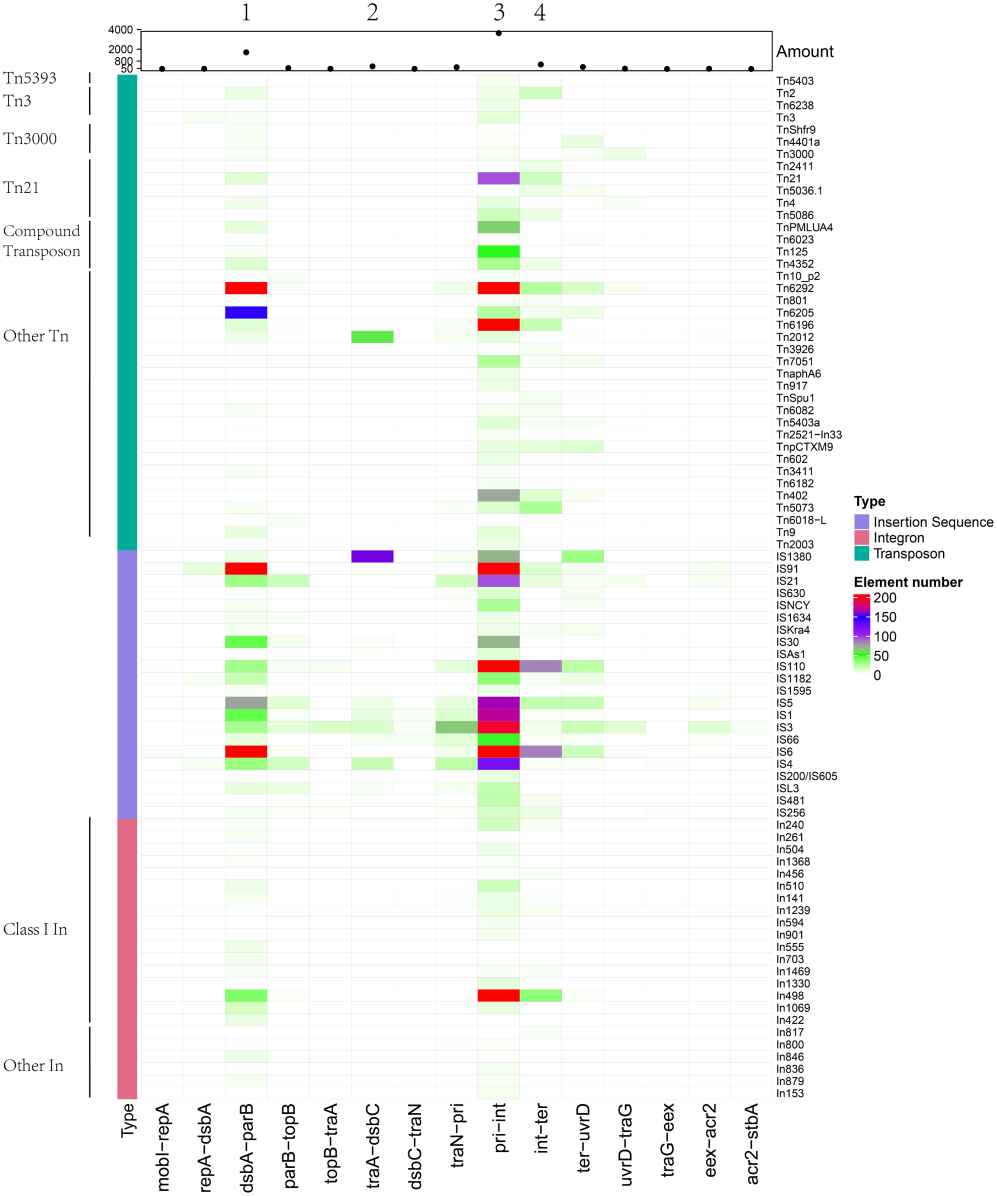
Heatmap of mobile genetic elements, including transposons, integrons and insertion sequences (amount > =3) in IncA and IncC plasmids. Their abundance is shown by the scatter plot, and group information is marked.

We also categorised MGEs into finer classifications and showed their distributions in the insertion spots. A total of 66 types of transposons, 58 types of integrons and more than 191 types of insertion sequences were found (Supplementary Table 5). We grouped transposons into Tn*163*, Tn*21*, Tn*3*, Tn*3000*, Tn*5053*, Tn*5393* and other groups and differentiated integrons into class I, II and other types and distinguished the insertion sequence into 22 IS families (Table 2). For transposons categorised into specific groups, Tn*21* and compound transposon were dominant distributed in insertion spot 3 and Tn*3* were primarily distributed in insertion spot 3 and insertion spot 4. Most of the integrons embedded in plasmids were class I, including In498, In240, In504, In1239, In510 and In846, In555, In703, In422, which were primarily distributed in insertion spot 3 and insertion spot 1, respectively. The major IS families were IS*110*, IS*6* and IS*91* in our analysed plasmids. For example, IS*4321*, IS*4321R* and IS*5075,* which belong to the IS*110* family, were widely distributed in insertion spot 3, and a number of IS*26* which belong to IS*6* family were inserted in insertion spot 1 and insertion spot 3 (Figure 7; Supplementary Table 5). Notably, the insertion of MGEs influenced the plasmid genes and led to the diverse structure of backbone. We found that MGEs carried other gene families of *int*, *traC, dsbC*. For example, various families of *int* were widely distributed in integrons and transposons as integrases. Transposons and insertion sequences, such as Tn*6292* carried *traC* and *dsbC* in pEc61B and pHS08-175-1, respectively, (Table 3). MGEs also interrupted core functional genes. We found gene fissions resulting from interruptions in genes involved in conjugative transfer (*traF*, *traU*, *traN*), DNA metabolism (*kfrA*) regulation (*acaD*) and other functions (*rhs, dsbC*; Table 3). Most gene fissions were induced by insertion sequences. For example, insertion sequences of the IS*3* family truncated *traF*, *traU*, *traN*, and *rhs,* and the IS*30* and IS*66* families insertion sequences truncated *rhs* and *traN, respectively.* And transposons also led to gene fissions. As Table 3 shows, Tn*5403a* induced the fission of *kfrA*. These results demonstrated that gene carriage and interruption by embedded MGEs contributed to the multiple forms and diversity of IncA and IncC plasmid genes, especially genes involved in essential systems. In addition, more than 41 classes resistances to antimicrobials were found in IncA and IncC plasmids such as beta-lactam, sulfonamide, and trimethoprim. As Supplementary Figure 5 shown, genes of anti-mercury, organomercury, streptomycin and sulfonamide significantly distributed in IncA and IncC plasmids though the number of IncA and hybrid IncA plasmids were less. The *blaTEM-1* gene tended to exist in type 2 IncC plasmids and *blaCMY-59* gene mainly distributed in type 1a and 1b IncC plasmids. In type 1a IncC, the number of genes involved in anti-mercury, chloramphenicol/florfenicol and sulfonamide were less than type 1b and 2 IncC plasmids. These differences may source from the divergent distribution of embedded MGEs and lead to different phenotypes to antibiotics in plasmids. However, further experimental works are still need to validate these resistance phenotypes and varied antimicrobials properties in different types of plasmids.

**Table 2:** Distribution of different groups/families of mobile genetic elements in the insertion insertion spots.

MGE groups/families	Insertion spot 1	Insertion spot 2	Insertion spot 3	Insertion spot 4	Summary
Tn*3*	10	0	22	19	51
Tn*21*	17	0	127	38	182
Tn*5053*	1	0	0	1	2
Tn*5393*	0	0	3	0	3
Tn*3000*	8	0	6	0	14
Tn*163*	0	0	1	0	1
Compound Transposon	24	0	145	6	175
Other Tn	501	56	690	114	1,361
IS*256*	4	0	14	7	25
IS*5*	75	6	165	20	266
IS*NCY*	3	0	27	0	30
IS*1634*	4	0	6	1	11
IS*630*	1	0	12	0	13
IS*4*	31	19	123	2	175
IS*As1*	1	0	10	0	11
IS*Kra4*	3	0	6	2	11
IS*200/IS605*	0	0	8	0	8
IS*1*	42	8	171	2	223
IS*66*	8	3	52	2	65
IS*1595*	2	0	7	0	9
IS*21*	30	0	94	8	132
IS*L3*	8	2	19	0	29
IS*30*	42	2	70	1	115
IS*481*	0	0	21	3	24
IS*91*	406	0	216	12	634
IS*1380*	7	156	71	0	234
IS*1182*	19	0	34	3	56
IS*6*	290	0	404	84	778
IS*3*	28	13	194	5	240
IS*110*	29	0	619	83	731
Class 1 integron	92	0	321	49	462
Class 2 integron	2	0	0	0	2
Other In	13	0	19	4	36

**Table 3:** Evolutionary events, including gene interruption and gene carriages, by mobile genetic elements.

Core functional gene	MGE	Plasmid number
*dsbC* ^a;b^	Tn*4352*,Tn*125*,Tn*6292*,IS*6*;IS*21*	4;1
*int* ^a^	In498,In879,In1069,In836,Tn*6375*,Tn*6187*,In1330,In571,In594	9
*traC* ^a^	Tn*6292*	1
*traU* ^b^	IS*3*	2
*traN* ^b^	IS*3*, IS*66*, IS*630*	6
*rhs* ^b^	IS*3*, IS*30*	11
*kfrA* ^b^	Tn*5403a*	1
*traF* ^b^	Tn*3411*, IS*3*	5
*acaD* ^b^	IS6	2

a. MGEs that carried core functional genes and the corresponding plasmid numbers. b. MGEs that interrupted core functional genes and the number of plasmids that experienced that evolutionary event.

### IncA and IncC plasmids are far more complex than their closest relative SXT/R391 ICEs

The SXT/R391 ICE is the closest known relative to the IncA and IncC plasmids. We therefore compared their shared key systems (Table 4). Their conjugative transfer system and several other core functional modules shared significant homology but with a wide range (Table 4). For genes in the conjugative transfer system, protein identity between IncA and IncC plasmids with SXT/R391 varied from 24.7 to 79.78%. For the conjugative module types, most SXT/R391ICEs comprised of MOB_H_ group relaxases and TrwB/TraD family T4CPs, which showed relative simplicity compared to IncA and IncC plasmids. All SXT/R391 ICEs contained only F-type T4SS genes, which suggests a monotonous constitution of their conjugative transfer system (Supplementary Table 6, 7). The recombination module (*ssb*, *bet*, and *exo*) in SXT/R391 and IncA and IncC plasmids shared 35.37% ~ 67.62% identity. In the entry exclusion system (*traG* and *eex*), the identity between *traG* from both MGEs was 29.92% ~ 44.78%, although the *eex* genes had no similarity (Table 4). Protein clustering results showed that families of nearly all core functional genes in IncA and IncC plasmids contained the corresponding gene families in SXT/R391 ICEs (Table 4). These findings indicated that the patterns of IncA and IncC plasmids in core functional genes nearly contained the varieties of SXT/R391 ICEs.

**Table 4:** Family number and similarities between shared core genes of IncA and IncC plasmids and SXT/R391 ICEs.

Shared core gene	Families number of IncA and IncC plasmids	Families number of SXT/R391 ICEs	Shared families number	Identity between two MGEs(%)	Coverage between two MGEs(%)
*mobI*	1	1	0	25.34–28.57	78.0–96.0
*traI*	5	2	1	24.7–52.23	51.0–87.0
*traD*	2	2	1	62.82–65.69	98.0–99.0
*traJ*	5	2	1	42.02–44.44	87.0–88.0
*traL*	3	1	1	61.43–64.52	98.0–100.0
*traE*	3	1	1	50.24–58.06	95.0–98.0
*traK*	3	1	1	43.51–45.61	91.0–91.0
*traB*	2	1	1	49.77–51.02	99.0–100.0
*traV*	3	1	1	43.58–48.41	67.0–87.0
*traA*	2	1	1	63.71–79.78	67.0–95.0
*traC*	3	1	1	50.63–51.26	98.0–99.0
*trhF*	1	1	1	45.18–52.9	93.0–99.0
*traW*	2	1	1	30.79–37.82	71.0–96.0
*traU*	2	1	1	45.51–65.71	84.0–99.0
*traN*	2	1	1	24.71–52.01	33.0–99.0
*ssb*	2	1	1	35.37–48.0	61.0–98.0
*bet*	1	1	1	58.49–67.62	76.0–91.0
*exo*	1	1	1	39.08–48.08	51.0–100.0
*int*	5	2	1	22.91–28.26	89.0–96.0
*traF*	2	1	1	39.87–45.91	79.0–91.0
*traH*	2	1	1	43.2–45.29	93.0–97.0
*traG*	4	1	1	29.92–44.78	49.0–99.0
*eex*	1	1	0	-	-

## Discussion

The present research systematically studied the genome characteristics and evolution of IncA and IncC plasmids based on large numbers of curated sequences. We found their core functional genes were occasionally deficient and existed as multiple functional copies/multiple families, which resulted in only one strictly core genes being found in all 678 plasmids and suggested that the core functional genes harboured a variety of diversity. Our results showed many multiple copies/multiple family genes originating from the carriage and interruption of MGEs embedded in plasmids. For example, TraF, a 347 amino acid protein in the pRM12581 plasmid, was disrupted by Tn*3411*, which resulted in two truncated genes with 79 aa and 271 aa, which was also noted in a previous study (Szabó et al., 2016). These results provided insights into the structural diversification of IncA or IncC plasmids and revealed that the distribution of MGE led to the diverse structure of backbone in IncA and IncC plasmids to some degree. Our analysis showed phylogeny of 13 core functional genes corresponded to IncA and IncC plasmids types well. Compared to the traditional typing method and current pMLST schemas, phylogeny based on concatenation of these genes provides a new approach to subtype IncA and IncC plasmids with optimum resolution, even plasmids with hybrid-type features (Hancock et al., 2017; Jolley et al., 2018).

Our findings demonstrated that the components of the conjugative transfer system in IncA and IncC plasmids were more complex than previously described. We found that IncA and IncC plasmids also contained MOB_C_, MOB_F_, MOB_P_, MOB_Q_ relaxases, VirD4/TraG T4CPs and P-type/I-type T4SS genes in the conjugative transfer system rather than the commonly depicted static gene clusters. Previous research found that the co-existence and adaptation of different MOB genes reformulated a new efficient transfer system (Garcillán-Barcia et al., 2019). Although complex and diverse types of conjugative transfer-related genes were found in IncA and IncC plasmids, the co-adaptation and functional compensation may maintain the system functions well indicating that classes of relaxase and T4CP could interact with different T4SS genes to reformulate a new transfer system. Notably, we found other types of conjugative transfer-related genes in the region near the integrases. For example, MOB_P_ relaxase, VirD4/TraG type T4CP and VirB-subtype T4SS genes were present in regions closest to integrase and other diverse embedded MGEs in plasmid pN13-01125 (Supplementary Tables 3, 5). This phenomenon suggested that these types of genes probably resulted from MGE-induced horizontal gene transfer. A previous report found a recombination site (RecHS) between the *rhs* and *int* genes in IncC plasmids (Hegyi et al., 2017). We found that some P-type/I-type T4SS genes were located in the region near RecHS, which indicated that homologous recombination may be a driving force for T4SS gene diversity in IncA and IncC plasmids.

Few of our analysed plasmids had identical backbones with the canonical description (Zhang et al., 2020). In contrast, we found that evolutionary events, such as core functional gene loss and duplication, led to backbone region deficiency and insertion. Genomic inversion induced by MGEs may reverse the order of certain backbone regions. This was supported by the IS*26*-induced inversions in the region from *parA* to *ter* (He et al., 2015; Mangat et al., 2017). The rearrangement between genomic regions also caused local disorder of the backbone, such as the fragment shift in plasmid p11298-tetA. The insertion of MGEs was predominantly present in 4 insertion spots. MGEs, such as Tn*21* and Tn*3* transposons, class I integrons and IS*110*, IS*6* and IS*91* IS families, were dominant in different insertion spots. Insertions varied between types and caused some of their type features, such as ARI-B. Possible recombination, such as previously mentioned RecHS-related events, may also lead to a variable backbone with multiple genes. Taking deletion, duplication, inversion, and rearrangement together, all of these evolutionary events led to the backbone of IncA and IncC plasmids being dynamically variable with diverse complexity and the diversity probably influenced the phenotype of plasmids, such as mobilization (Wang et al., 2019). The considered closest relatives of IncA and IncC plasmids, the SXT/R391 family integrative conjugative element (ICE) was comprised only of a relatively simple constitution of conjugative transfer systems, such as MOB_H_ group relaxases, TrwB/TraD family T4CPs, and F-type T4SS genes. Most shared genes between both MGEs had considerable identity, and the families of shared core functional genes were nearly contained by genes from IncA and IncC plasmids. Our comparison revealed that the IncA and IncC plasmids were more diverse, and the SXT/R391 ICEs were simpler. The complexity of the IncA and IncC plasmids included the possibility of SXT/R391 ICEs in shared parts.

In summary, compared to exact case studies, our approach provided a global and systematic view of IncA and IncC plasmids. We found that most core functional genes were not strictly conserved, occasionally deficient and sometimes existed as multiple copies/multiple families. We also found the phylogenetic relationship of some core functional genes that corresponded to the types of plasmids could be used to supplement the plasmid classification. As an important module of the backbone, we found that the conjugative transfer system was more complex than the current understanding of gene clusters. The insertion of MGEs varied between plasmid types and presented in 4 insertion spots with certain types of transposons, integrons and insertion sequences. Due to the impact of gene duplication and loss, insertion of MGEs, genome rearrangement and recombination, the backbones of the IncA and IncC plasmids were actually dynamic, variable and diverse. The IncA and IncC plasmids were more complex than their closest relative SXT/R391 ICEs and included nearly all of the diversity of SXT/R391 ICEs in key systems. However, our study was also limited by the source countries, organism and types of plasmids. A significant part of plasmids was isolated from *Escherichia coli* or *Klebsiella pneumoniae*. The majority of plasmids were originated from China and America and few IncA plasmids were included in the study. An ever-increasing sequence data of IncA and IncC plasmids from all over the world will strengthen our findings and provide more insights into their evolutionary history and complex genomic features. Additionally, the diverse conjugation-related genes and embedded MGEs in plasmids were analysed using homology-based prediction. Their biological function and influence to plasmids evolution and phenotype were still unclear. Therefore, further validation is required. Overall, our findings shed new light on its genome evolution and provide valuable new insight into its complex characteristics.

## Materials and methods

### Plasmid collection, core functional gene recognition and phylogeny

We manually collected IncA and IncC plasmid accessions from some databases including Refseq database,^1^ NCBI’s prokaryotic genome database,^2^ the PLSDB database (Schmartz et al., 2022), a comprehensive database of plasmid sequences (Brooks et al., 2019) and others.^3^,^4^ An Entrez query was performed to retrieve as many Refseq genomic sequences of these plasmids as possible from the GenBank nucleotide sequences database. After removing incomplete and redundant sequences, we searched the replicons from PlasmidFinder using the Enterobacteriaceae and the Gram-positive datasets with default parameters (Carattoli et al., 2014; Orlek et al., 2017). We curated a total of 678 plasmid sequences and their meta-information (Supplementary Table 1). The subtype of the IncC plasmids were identified (Harmer and Hall, 2017; Ambrose et al., 2018b). Beside IncA, type 1a IncC, type 1b IncC and type 2 IncC plasmids, we defined the plasmids having other types of replicons as hybrid-type IncA or IncC. The IncC plasmids that could not be attributed to concrete types were regarded as untyped IncC plasmids (Orlek et al., 2017). All plasmid sequences were re-linearised using the mobI gene as the beginning. Core functional genes were identified with > = 50% identity and coverage and a 0.001 e-value threshold (Zou et al., 2019; Zhou et al., 2020). The identified core functional genes were assigned to orthogroups using OrthoFinder clustering analysis, which uses all-versus-all BLAST and MCL approaches (Emms and Kelly, 2019). CD-HIT was used to reduce sequence redundancy with identity 100% (Fu et al., 2012). Phylogenetic trees of 43 core functional genes and a concatenated tree of the genes *repA, dsbA, sppA, parA, topB, traI, traD, traC, trhF, traW, stbA, traN* and *pri* were inferred with selected sequences of plasmids containing these genes using the maximum likelihood method in MEGA (Tamura et al., 2021). The amino acid divergence of core functional genes between different types of plasmids was analysed by mapping back to plasmid pR55, the reference plasmid of type 2 IncC plasmids which were the primarily type in our dataset (Doublet et al., 2012).

### Prediction of conjugative transfer systems

All related genes in conjugative transfer systems of plasmids were predicted by a thorough homologous search with genes in the oriTDB (Li X. et al., 2018) and SecRet4 databases (Bi et al., 2013; Hu et al., 2021). Relaxases, T4CPs and MPFs/T4SS genes in plasmids were predicted with identity > = 50, coverage > = 50% and e-value <=0.001. Relaxase can be divided into six types (MOB_H_, MOB_F_, MOB_C_, MOB_P_, MOB_Q_ and Other), and T4CPs were distinguished into the VirD4/TraG subfamily, TrwB/TraD subfamily and Other (Li X. et al., 2018). T4SSs and related genes in plasmids were classified into types F, P and I and further distinguished into different sub-families, such as Trb, Trw, VirB, Tra_I and Dot/Icm. Plascad was used to classify plasmids into three categories: conjugative, mobilizable and non-mobilizable (Che et al., 2021). A gene content tree was constructed using all identified T4SS protein sequences of plasmids with high genomic completeness. The tree was visualised and annotated with the information of T4SSs, conjugative type, relaxases and T4CPs.

### Mobile genetic elements and antimicrobial resistance genes in plasmid variable regions

Integrons in plasmids were predicted using sequences in the INTEGRALL database, which contains more than 4,800 integron sequences and provides a public genetic repository for sequence data and nomenclature (Moura et al., 2009). Transposons were identified with sequences in the Transposon Registry, which is a system containing transposons from bacterial and archaeal autonomous transposable elements (TEs), including unit transposons, composite transposons, conjugative transposons (CTns)/integrative conjugative elements (ICEs), mobilizable transposons (MTns)/integrative mobilizable elements (IMEs) and mobile genomic islands (Tansirichaiya et al., 2019). Genes conferring resistance to antimicrobials were identified using sequence in NCBI and CARD database (Alcock et al., 2020), which is a comprehensive antibiotic resistance database providing reference DNA and protein sequences. Sequence searches were performed using BacAnt with identity > = 90% and coverage > = 60% (Hua et al., 2021). We also expanded the transposon database in BacAnt using data from TnCentral, which currently contains ~400 carefully annotated TEs, including transposons from the Tn*3*, Tn*7*, Tn*402*, and Tn*554* families, compound transposons and integrons (Ross et al., 2021). Insertion sequences in plasmids were searched using ISfinder (Siguier et al., 2006) with a threshold of identity and coverage ≥50% and e-value <0.001.

### Dynamic plasmid backbone annotation

To comprehensively describe the backbone of all IncA and IncC plasmids, we compared all analysed plasmids with the reference plasmid pR55. All plasmid genes were re-linearised using the mobI gene as the beginning. If a core functional gene was absent in a plasmid compared to the classical backbone position in pR55, this absence was annotated as a gene deletion. If a core functional gene appeared more than once other than in the classical backbone position of pR55, other copies of the gene were annotated as duplications. When a continuous gene fragment was in a reverse order compared to pR55, this condition was annotated as inversion. If there were position exchanges of genomic segments in plasmids, we depicted it as a rearrangement (Ma et al., 2017). All evolutionary events, such as deletion, duplication, inversion and interchange, were summarised and depicted in addition to the classical static backbone.

### Comparison of IncA and IncC plasmids with SXT/R391 ICEs

A total of 45 complete SXT/R391 ICEs were collected to comparison after screening (Liu et al., 2019). We obtained the distribution of common core genes in SXT/R391 ICEs *via* homology searching. The components of the conjugative transfer system were identified, and clustering analysis was performed. We subsequently compared the types of components in the conjugative transfer system in IncA and IncC plasmids and SXT/R391 ICEs and families of other core functional genes. We computed the similarities of their shared gene families from key modules, such as conjugative transfer (*mobI*, *traI*, *traD*, *traL*, *traE*, *traK*, *traB*, *traV*, *traA*, *traC*, *trhF*, *traW*, t*raU*, *traN*, *traF*, *traH*, and *traG*), recombination (*ssb*, *bet*, and *exo*), entry exclusion (*eex*), and DNA metabolism (*int*), in both MGEs.

## Data availability statement

The original contributions presented in the study are included in the article/Supplementary material, further inquiries can be directed to the corresponding authors.

## Author contributions

JY and YJ conceived the study and designed the experimental procedures. WL and BL collected the information. MH and HR did the phylogenetic analysis. FZ, XY, BW, and ZY analysed the all genomic features and evolutionary characteristics of the plasmids. JY, YJ, and FZ drafted and revised the manuscript. All authors contributed to the article and approved the submitted version.

## Funding

This study was supported by funds from the National Natural Science Foundation of China (Nos. 31801096 and 31671363), Research Project from State Key Laboratory of Pathogen and Biosecurity (No. SKLPBS1813), and National Key Research and Development Program of China (No. 2022YFC2600704).

## Conflict of interest

The authors declare that the research was conducted in the absence of any commercial or financial relationships that could be construed as a potential conflict of interest.

## Publisher’s note

All claims expressed in this article are solely those of the authors and do not necessarily represent those of their affiliated organizations, or those of the publisher, the editors and the reviewers. Any product that may be evaluated in this article, or claim that may be made by its manufacturer, is not guaranteed or endorsed by the publisher.

## Footnotes

^1^ https://ftp.ncbi.nlm.nih.gov/genomes/refseq/plasmid/

^2^ https://www.ncbi.nlm.nih.gov/genome/browse#!/prokaryotes/

^3^ https://ftp.ncbi.nlm.nih.gov/genomes/GENOME_REPORTS/IDS/Plasmids.ids

^4^ https://ftp.ncbi.nlm.nih.gov/genomes/archive/old_refseq/Plasmids/Plasmids.ids

## Supplementary material

SUPPLEMENTARY FIGURE 1 Phylogenetic relationship of IncA and IncC plasmids containing the 13 genes and corresponding amino acid divergence. The evolutionary history was inferred using concatenated core functional genes. The difference in amino acids compared with the reference plasmid pR55 is shown by coloured circles that represent different amino acids.

SUPPLEMENTARY FIGURE 2 Phylogenetic relationship of IncA and IncC plasmids inferred using gene *sppA*.

SUPPLEMENTARY FIGURE 3 Phylogenetic relationship of IncA and IncC plasmids inferred using gene *topB*.

SUPPLEMENTARY FIGURE 4 Phylogenetic relationship of IncA and IncC plasmids inferred using concatenation of gene *sppA* and *topB*.

SUPPLEMENTARY FIGURE 5 Distribution of genes conferring resistance to antimicrobials in IncA and IncC plasmids.

SUPPLEMENTARY TABLE 1 Detailed information on the 678 IncA and IncC plasmids included in this research.

SUPPLEMENTARY TABLE 2 The orthogroup clusters of core functional genes in all plasmids.

SUPPLEMENTARY TABLE 3 The conjugative types of plasmids and the relaxase, T4CP and T4SS genes contained in plasmids.

SUPPLEMENTARY TABLE 4 The deletion, duplication of genes or gene clusters, inversion and rearrangement in all plasmids and the arrays of backbone genes.

SUPPLEMENTARY TABLE 5 Presence and absence of the insertion MGEs in plasmids.

SUPPLEMENTARY TABLE 6 Meta-information of 45 SXT/R391 ICEs used to study the relationship with IncA and IncC plasmids.

SUPPLEMENTARY TABLE 7 Types of relaxases and T4CPs and T4SS genes in the conjugative transfer system of all SXT/R391 ICEs.
