# Supplementary figures and images for: Comparative genomics reveals new insights into the evolution of the IncA and IncC family of plasmids
Source: Front Microbiol. 2022 Nov 16;13:1045314. doi: 10.3389/fmicb.2022.1045314 (PMC9709138; doi:10.3389/fmicb.2022.1045314)

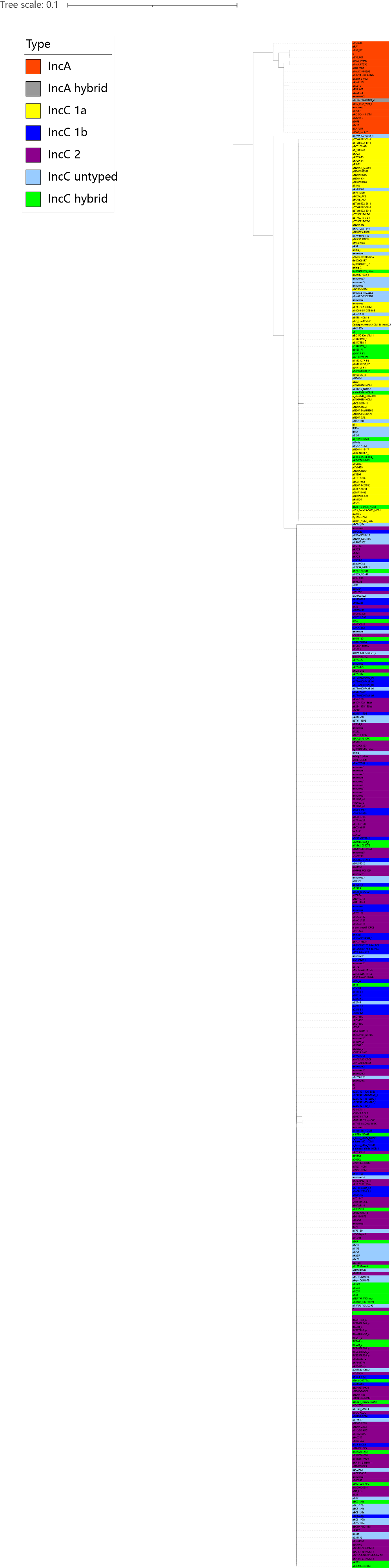

Supplement: SUPPLEMENTARY FIGURE 2 — Phylogenetic relationship of IncA and IncC plasmids inferred using gene sppA. [file Image_2.TIF]

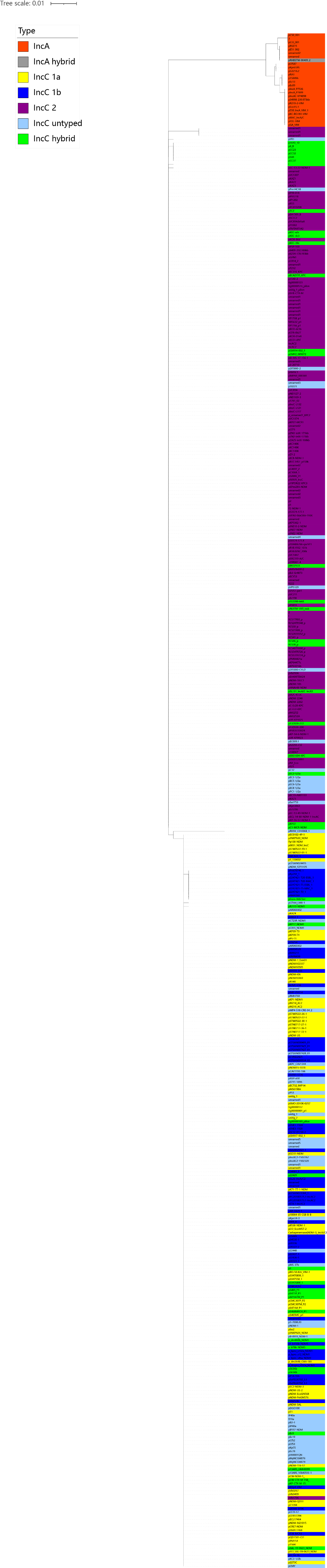

Supplement: SUPPLEMENTARY FIGURE 3 — Phylogenetic relationship of IncA and IncC plasmids inferred using gene topB. [file Image_3.TIF]

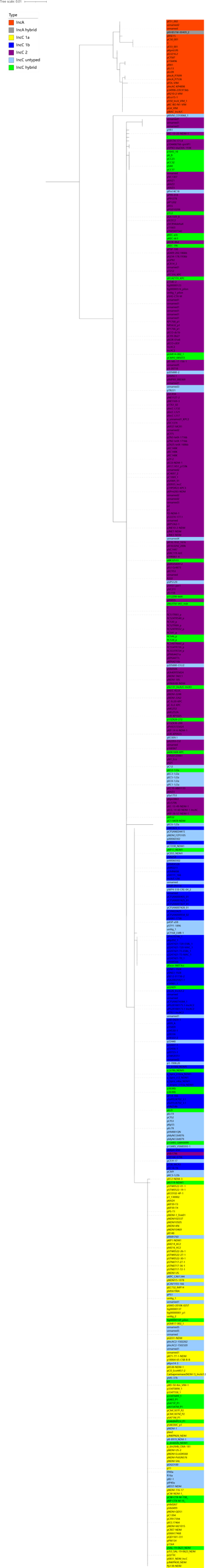

Supplement: SUPPLEMENTARY FIGURE 4 — Phylogenetic relationship of IncA and IncC plasmids inferred using concatenation of gene sppA and topB. [file Image_4.TIF]

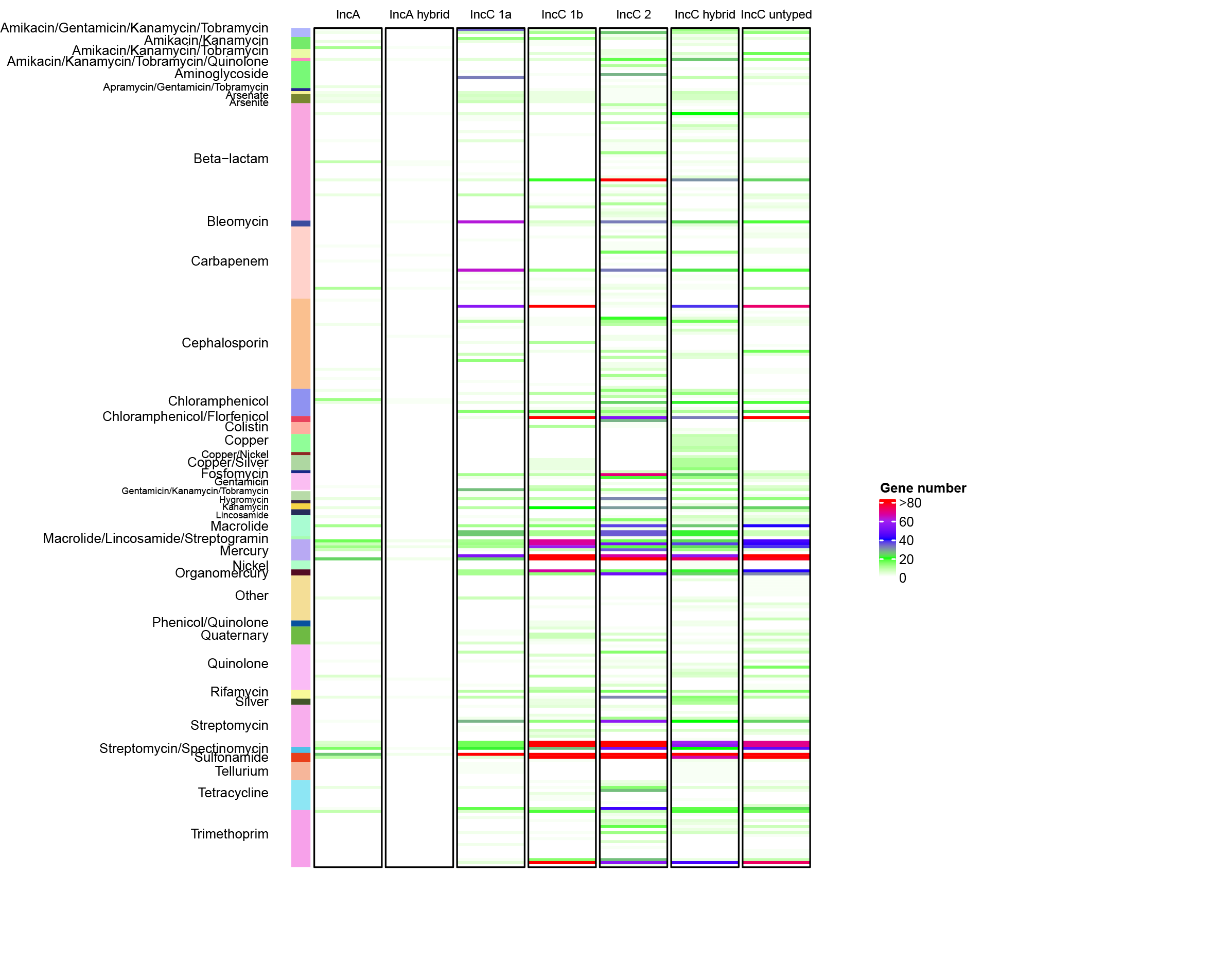

Supplement: SUPPLEMENTARY FIGURE 5 — Distribution of genes conferring resistance to antimicrobials in IncA and IncC plasmids. [file Image_5.TIF]
